# Investigating habits: strategies, technologies and models

**DOI:** 10.3389/fnbeh.2014.00039

**Published:** 2014-02-12

**Authors:** Kyle S. Smith, Ann M. Graybiel

**Affiliations:** ^1^Department of Psychological and Brain Sciences, Dartmouth CollegeHanover, NH, USA; ^2^Department of Brain and Cognitive Sciences, McGovern Institute for Brain Research, Massachusetts Institute of TechnologyCambridge, MA, USA

**Keywords:** striatum, cortex, action, chunking, learning, flexibility, reward, reinforcement

## Abstract

Understanding habits at a biological level requires a combination of behavioral observations and measures of ongoing neural activity. Theoretical frameworks as well as definitions of habitual behaviors emerging from classic behavioral research have been enriched by new approaches taking account of the identification of brain regions and circuits related to habitual behavior. Together, this combination of experimental and theoretical work has provided key insights into how brain circuits underlying action-learning and action-selection are organized, and how a balance between behavioral flexibility and fixity is achieved. New methods to monitor and manipulate neural activity in real time are allowing us to have a first look “under the hood” of a habit as it is formed and expressed. Here we discuss ideas emerging from such approaches. We pay special attention to the unexpected findings that have arisen from our own experiments suggesting that habitual behaviors likely require the simultaneous activity of multiple distinct components, or operators, seen as responsible for the contrasting dynamics of neural activity in both cortico-limbic and sensorimotor circuits recorded concurrently during different stages of habit learning. The neural dynamics identified thus far do not fully meet expectations derived from traditional models of the structure of habits, and the behavioral measures of habits that we have made also are not fully aligned with these models. We explore these new clues as opportunities to refine an understanding of habits.

There is a distinguished history of scientific attention to habitual behaviors. In early thinking in psychology, much of behavior was framed in terms of lack of mindfulness, and this mode of behavior was considered as habitual. This was, and remains, a straightforward and intuitive way of thinking. This line of thinking was convolved with the classic reflex arcs of Sherrington and his students, the ubiquity of habits emphasized by William James, the “law of effect” of Thorndike, the formalized drive theory of Hull, and with the general behaviorist research tradition. As a consequence, many aspects of animal behavior were viewed in terms of sensory inputs and movement outputs linked by what we would now call neural networks (James, 1890; Thorndike, 1898; Sherrington, 1906; Hull, 1943). As ethologic approaches developed, views emerged emphasizing that species-specific instinctual behaviors are characterized by a consistency in performance similar to, or exceeding, that of learned behaviors (Tinbergen, 1950; Lorenz and Leyhausen, 1973). It now is clear that even so-called simple reflex arcs lie within modifiable microcircuits (Marder, 2011), as already forecast in the work of Sherrington and embedded in the ideas of William James.

In other fields, also, there has been pushback against the idea that animals other than humans behave without some form of mindfulness. Affective and incentive motivational processes have been seen as guiding forces for behavior as potent as stimulus-response (S-R) associations (Bolles, 1972; Bindra, 1978; Toates, 1986; Berridge, 2004; Salamone and Correa, 2012). Many features of behavior have been identified as being driven actively as intentional processes, rather than by habit (Tolman, 1932; Holland, 2008). Habits were, accordingly, sometimes separated from mainstream fields investigating behavior, especially cognitive decision-making behavior. Now, in part due to new methods emerging to approach this issue more directly, an active field is exploring what habits might be and what they might not be (Dickinson, 1985; Graybiel, 1997, 2008; Daw et al., 2005a; Yin and Knowlton, 2006; Wood and Neal, 2007; Holland, 2008; Redish et al., 2008; Belin et al., 2009; Packard, 2009; Balleine and O’Doherty, 2010; Berridge and O’Doherty, 2013; Dolan and Dayan, 2013).

Here we summarize the results of experiments in which we asked whether we could, by employing optogenetic methods, manipulate habitual behaviors—either changing them after they had formed, or preventing them from being formed. This attempt at gaining causal evidence about the control of habits suggests that they are not always stand-alone S-R behaviors, but rather, can be behaviors that are carefully monitored and controlled by neural circuits on-line in real time despite their apparent automaticity. We suggest that habits can be characterized as constituting sequences of actions that have been chunked together through simultaneous cortical and basal ganglia activity dynamics and as arising from multiple core operators in the brain that control habits in real time. Our intent here is to focus on the results and implications of newer optogenetic and recording studies, but we encourage readers to compare and contrast recent articles for comprehensive views on brain mechanisms related to learned actions and habits (Graybiel, 2008; Balleine et al., 2009; Packard, 2009; Balleine and O’Doherty, 2010; Dolan and Dayan, 2013).

## Operational definitions and measures of habitual behavior

Studies of habitual behaviors use a range of contemporary empirical approaches to probe their structure in species ranging from flies to rodents to humans (Dickinson, 1985; Yin and Knowlton, 2006; Brembs, 2009; Tricomi et al., 2009; Balleine and O’Doherty, 2010). We categorize a few of these approaches here, and the criteria they set up for definitions of habitual behavior, but the list that we give is not exhaustive; nor is each measure exclusive of others. Even within single tasks, key task demands can push behavior either into more mindful, goal-directed, flexible modes, or into more habitual, repetitive or fixed modes (Adams, 1982; Dickinson, 1985; Daw et al., 2005a; Yin and Knowlton, 2006; Balleine et al., 2009; Packard and Goodman, 2013). For example, in order to encourage habits, protocols often include extended and distributed training, increased reward exposure, diluted contingency between actions and rewards or increased informational uncertainty, and reduction in reward contiguity with sensory inputs. The fact that habitual and cognitively driven behaviors can vary inversely when task conditions change (or when experimental brain lesions are imposed) suggests that there is likely to be a competitive or at least parallel architecture in neural circuits driving habitual and goal-directed expression of behavior, a point much emphasized in current theoretical work (Dickinson, 1985; Balleine and Dickinson, 1998; Killcross and Coutureau, 2003; Daw et al., 2005b; Packard and Goodman, 2013) (but also see Dolan and Dayan, 2013).

### Outcome representation

In a clever extension of the early logic that S-R and non-S-R modes of behavior might both exist, revaluation of the reinforcement outcome offered was introduced into an operant task in order to pit the two behavioral strategies against one another (Adams, 1982; Dickinson, 1985; Dickinson and Balleine, 2009). Logic held that if behaviors were driven more by “cognitive” associations between an action and a particular expected outcome (goal-driven behavior), then changing the value of the expected outcome should directly influence the action. Conversely, if a behavior were like an inflexible S-R reflex lacking outcome representation, then outcome changes ought to not affect it.

In an early experiment testing this logic (Adams, 1982), animals were trained on a simple operant task (e.g., press a lever for reward). They then received a devaluation of the reward, which involved induction of conditioning an aversion to the reward through pairings of the reward with administration of a nauseogenic agent, lithium chloride. In this paradigm, once the reinforcement is experienced in its newly devalued state, and knowledge about the value reduction is acquired, animals are returned to the task and tested in a probe trial in which no reinforcement is present—so-called extinction conditions. Animals that have been trained in the task, but have not been trained far past their initial acquisition performance levels, reduce their performance of the action that was paired with obtaining the newly devalued reward. This finding suggested that the animals recognize the identity of the outcome that they are working for, and can flexibly adjust whether they will or will not work to receive it when its subjective value changes, providing support for earlier notions of animal purposefulness in behavior (Tolman, 1932; Tolman and Gleitman, 1949; Holland, 2008; Dickinson and Balleine, 2009). By contrast, animals that have been over-trained on the task, that is, trained over and over long after they reached an initial learning criterion, will continue working for it. This persistence of seeking the reinforcement despite its devaluation is considered to be a defining mark of habitual behavior.

Over-training is, however, not necessary for such habitual behavior. Habits can also form in animals without extended training if they are trained on variable interval task schedules (rather than on ratio schedules) (Dickinson et al., 1983). In these variable interval schedules, a reward is given after a varying length of time, once the initial task behavior is established, in order to dilute directly the contingency between each action and each reward experience (Dickinson, 1985; Balleine and Dickinson, 1998). Thus, an additional criterion for defining a behavior as habitual emerged from this line of work, by which animals should be insensitive both to outcome value as well as to changes in the consistency of the action-outcome (A-O) contingency (Dickinson, 1985; Balleine and Dickinson, 1998).

Among associative learning psychologists, these two instrumental learning constructs are contrasted to Pavlovian stimulus-stimulus (S-S) learning, because this form of learning (as in bell indicates food) tends to be directly sensitive to reward revaluation (Holland and Rescorla, 1975; Balleine and Dickinson, 1992; Dickinson and Balleine, 2009) and generally insensitive to A-O contingencies (Williams and Williams, 1969; Stiers and Silberberg, 1974; Hershberger, 1986) (see also below for exceptions).

**Figure 1:**
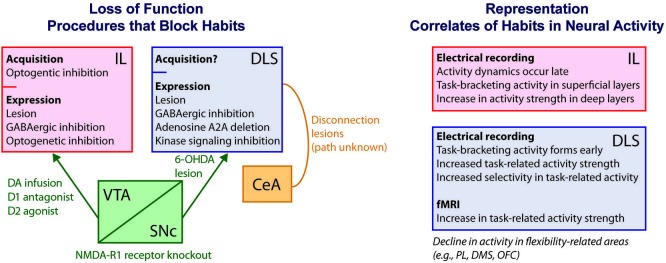
**Schematic of known habit-related mechanisms using measures of behavioral outcome-sensitivity**. At left, diagram of loss-of-function results in which habits are suppressed or blocked following neural interventions. The IL cortex is viewed as necessary for habit expression due to greater outcome-sensitivity in behavior resulting from lesions, temporary pharmacologic or optogenetic inhibition, or manipulations of dopamine-containing input including intra-IL infusion of dopamine, a D1 antagonist, or a D2 agonist (Coutureau and Killcross, 2003; Killcross and Coutureau, 2003; Hitchcott et al., 2007; Smith et al., 2012; Barker et al., 2013). The DLS is similarly needed for habit expression, as demonstrated through lesions, chemical inhibition, molecular signaling inhibition, gene deletion, and dopaminergic denervation (Yin et al., 2004, 2006; Faure et al., 2005; Yu et al., 2009; Gourley et al., 2013;). Burst firing of dopaminergic neurons is also needed, demonstrated by NMDA-R1 receptor knockout (Wang et al., 2011). Interaction between the CeA and the DLS, through unknown anatomical routes, is required as well (Lingawi and Balleine, 2012). Although lesions confound acquisition and expression phases of habits, a role for IL in habit acquisition specifically has been shown using optogenetics (Smith and Graybiel, 2013a). At right, diagram and list of some key features of IL and DLS neural activity related to habit formation and expression as uncovered from electrical recording and fMRI approaches (Tang et al., 2007; Tricomi et al., 2009; Thorn et al., 2010; Gremel and Costa, 2013; Smith and Graybiel, 2013a). Concomitant to these dynamics are a decline in activity in areas that might oppose habits, including the PL, DMS, and OFC (Thorn et al., 2010; Gremel and Costa, 2013; Smith and Graybiel, 2013a). IL, infralimbic cortex; DLS, dorsolateral striatum; VTA, ventral tegmental area; CeA, central nucleus of the amygdala; PL, prelimbic cortex; DA, dopamine; DMS, dorsomedial striatum; OFC, orbitofrontal cortex.

A large number of loss-of-function neuroscience experiments supports this framework for interpreting habits as outcome-insensitive S-R near-reflexes (Figure 1). For example, normally outcome-insensitive behaviors can be rendered outcome-guided following disruption (through lesions, chemical inactivation, or gene knockout) of the sensorimotor striatum (called the dorsolateral striatum or DLS). Similar effects are found after disruption of the dopamine-containing input to the DLS from the *pars compacta* of the substantia nigra, after disconnection of the DLS and the central nucleus of the amygdala (indirectly connected), or after disruption of pallidum-projecting neurons in the striatum in general (Yin et al., 2004; Faure et al., 2005; Yu et al., 2009; Wang et al., 2011; Lingawi and Balleine, 2012). Disruption of the infralimbic subdivision of medial prefrontal cortex (here called the infralimbic (IL) cortex) produces similar effects (Coutureau and Killcross, 2003; Killcross and Coutureau, 2003; Hitchcott et al., 2007; Smith et al., 2012; Barker et al., 2013). The fact that the IL cortex is not directly connected with the DLS, along with the many other regions implicated, suggests a widespread distribution of habit-promoting regions in the brain.

### Multiple memory systems

Related to this line of thinking is the notion that behaviors in environments requiring navigation (e.g., mazes) can be guided by spatial cues, or instead can be driven by learned movement plans. In one famous test, a so-called plus maze was used to tease apart such “place” (allocentric) and “response” (egocentric) strategies (Tolman et al., 1947; Packard and McGaugh, 1996). Animals might start at the south arm and be required to turn east (right) to receive reward. After a training period, animals then would be started in the north arm. If they followed the spatial cues, as trained rats often do, they would turn left to enter the east arm. If, instead, they had learned to make a particular response, as over-trained animals might do, they would turn right to enter the west arm. Habitual behavior, according to this work and its precedents, was identified by the adoption of such a response-based maze running strategy. In lesion work, the DLS and dopamine have once again been implicated as required for such a habitual strategy (McDonald and White, 1993; Lee et al., 2008; Packard, 2009; Wang et al., 2011).

### Learned response vs. tree-search

Tasks also are often designed to assess the acquisition and persistence of stimulus-evoked responses, such as in having animals learn to respond in one way to a specific cue in order to receive a reward and to use various decision-making strategies that are more or less cognitive or habitual. Recent computational work has conceived of habits as resulting from model-free learning systems in the brain (Daw et al., 2005a; McDannald et al., 2012; Dolan and Dayan, 2013). In this view, behavior is ultimately brought under the control of two systems—one forward-looking (model-based), and one that stores value based on experience (model-free). Model-free control, analogous to habitual behavioral control, is thought to capture a multitude of behavioral phenomena and corresponding neural findings, the idea being that behaviors that have been stored through prediction-error learning might share a common computational structure (Doya et al., 2002; Daw et al., 2005a; Dolan and Dayan, 2013). This framework of learning covers traditional instrumental and Pavlovian conditioning realms, in which instances of both model-free and model-based control is thought to occur (Berridge and O’Doherty, 2013; Dolan and Dayan, 2013), and has been used to positive effect in work on brain function in both rodents and humans (Daw et al., 2005a; Bornstein and Daw, 2011; McDannald et al., 2012; Dolan and Dayan, 2013). The emerging views are at a stage of vigorous debate. Lines of evidence have arisen that support the existence of dissociable biological substrates for each system, for competition between them, for toggling between them in single behaviors, for merging them, and even for model-based systems teaching model-free systems as collaborators (Dolan and Dayan, 2013). Nonetheless, once again, such work has implicated the striatum, dopamine, and particular circuits in the neocortex in controlling the acquired model-free behavioral plan (Daw et al., 2005a; McDannald et al., 2012; Dolan and Dayan, 2013).

### Performance optimization

Reminiscent of early thinking is the idea that habits are behaviors that have become well-practiced, routine, and predictable. Useful hallmarks for the formation of skills and habit-like behaviors include increased speed to start and complete tasks, more stereotypic and routed movements through a task environment, fewer deliberations at decision points, reduced distractibility, indifference to negative feedback, and increased performance accuracy (Poldrack et al., 2005; Graybiel, 2008; Belin et al., 2009; Desrochers et al., 2010; Hikosaka and Isoda, 2010; Smith and Graybiel, 2013a). Such measures do not distinguish the possible covert strategies or task representations that might be controlling behavior; but they do provide an important set of reference points for understanding a behavior as more or less exploratory and might serve as reference points for understanding the progression of habit formation and the involvement of potential brain mechanisms.

## Neurophysiological correlates of habitual action learning

We turn now to insights gained from exploiting relatively recent technological advances, including chronic recording from behaving animals and gene-based targeting strategies for manipulating brain activity in real time by optogenetics. Loss-of-function studies, such as those highlighted above, provided strong evidence for regions of the neocortex and basal ganglia as being critical for the expression of habits. It is possible at some level to map these roles onto changes in neural activity recorded from these regions. However, they have not yet converged on a unified view of the underlying structure of a habitual behavior. This difference is the main focus of our discussion.

Numerous changes in neuronal activity in the neocortex and basal ganglia have been documented in rodents and primates as learned behaviors shift with practice from exploratory to skillful in their execution. Ensembles of neurons develop time-locked responses to well learned or innate sequences of actions such as grooming patterns or sequences of motifs in birdsong, often with distinct representations of distinct steps within a sequence (Berns and Sejnowski, 1998; Brainard and Doupe, 2002; Fujii and Graybiel, 2003, 2005; Aldridge et al., 2004; Jin et al., 2009; Hikosaka and Isoda, 2010; Fee and Goldberg, 2011). Related work in rats has focused on the plasticity of somatotopic representations in the striatum during motor learning. For example, by recording the activity of forelimb-sensitive neurons in rats performing a forelimb reaching task, Carelli and West found that as the movements were repeated over time for reward, there was a decline in the response of movement-related neurons (Carelli et al., 1997; Tang et al., 2007). This result points to a contrast with the findings in cortical recording experiments on related tasks (Karni et al., 1995; Nudo et al., 1996; Plautz et al., 2000); instead of increasing the representation of movements as they are put to use in a task, the striatum appears to become more selective or efficient in such representations. This work raised the important possibility that regions needed for habit expression are themselves key sites of neuroplasticity. The way in which these plasticity changes reshape neural ensemble activity has been surprising.

### As the dorsolateral striatum (DLS) sees habits: action chunking representations

We have explored this plasticity in striatal activity in the DLS region targeted in the loss of function studies, by recording for the entire time during which rats and mice develop habitual behaviors with extensive training on T-maze tasks (in which reward is obtained following correct navigation in response to instruction cues) (Figure 1). The findings in these studies have provided evidence for viewing habits fundamentally as sequence of actions that are grouped together, or “chunked”, for ready deployment. By recording simultaneously from many putative medium-spiny neurons (MSNs) in the DLS day by day, we asked what neural activity might occur during task training and over-training.

An initial study (Jog et al., 1999) documented a remarkable change in patterning of spike activity in the DLS viewed in the framework of entire run-times. In the relatively naïve animals, DLS ensembles were active during the runs, but as training continued and the runs became well-practiced and faster, many individual units in the DLS ensembles developed activity that was particularly pronounced at the beginning and/or at the end of the runs, and fewer were active mid-run. This beginning-and-end task-bracketing activity provided a compelling candidate neural correlate for the chunking of actions together into a habitual unit (Graybiel, 1998). It has long been noted that individual elements of memories can be chunked together to aid recall (Miller, 1956)—as we recall PIN numbers, passcodes, and phone numbers. It is difficult to remember the elements other than within the whole, as witnessed by the trouble we have in picking up in the middle after interruptions in recall. In the context of actions, plausibly, a well learned action-sequence could similarly be chunked into a performance unit. The recorded DLS activity certainly appeared to reflect this process and suggested indeed that a habit might in part be a chunked-together sequence of behavioral steps.

It soon became clear that not only the numbers of neurons, but also the spike rates of the neurons, contributed to the task-bracketing patterns (Barnes et al., 2005). Moreover, the chunking pattern grew as the animals learned the habit, but changed if conditions changed during extinction and reinstatement tests (Barnes et al., 2005). When the rewards presented at the goal sites during the training phase were then removed or greatly reduced, the DLS bracketing pattern returned to roughly the same pan-run pattern present in the untrained animals, and when the rewards were suddenly returned, the bracketing pattern returned in nearly its fully developed form. Was the neural activity pattern suppressed? Passed to another brain region? Too small to detect with extracellular ensemble recording methods? These are very live issues that need answers. What these dynamics do suggest is that just as habits, once developed, are difficult to forget, so the patterned neural activity that accompanies them can be suppressed but still is maintained so that it can be expressed rapidly when conditions call for it (Pavlov, 1927; Rescorla, 1996; Barnes et al., 2005). The DLS dynamics appear to play a key role in this rapid loss and recovery of learned habits.

This bracketing pattern has since been found in other regions as well. It emerges as part of the neural representation of song repetition in HVC of Bengalese finches (Fujimoto et al., 2011), in primate prefrontal cortex as a series of saccades are performed by highly trained monkeys (Fujii and Graybiel, 2003), and in the substantia nigra of mice as they learn to press a lever repeatedly for reward (Jin and Costa, 2010). The DLS “end-related activity” can phasically precede or follow the end of a performance sequence, or appear as phasic activity around finalizing actions, such as turning in the T-maze task, depending on task-type or potentially the trial-to-trial time-locking of repeated actions for analysis (Barnes et al., 2005; Kubota et al., 2009; Jin and Costa, 2010; Thorn et al., 2010; Smith and Graybiel, 2013a).

Within the DLS, the chunking pattern is not restricted to the MSNs, but can be expressed by interneurons as well, but with interesting differences. Kubota et al. (2009) trained and then over-trained mice in the standard maze T-maze task in which turn directions were instructed by auditory cues, and then without warning switched the modality of the instruction cues from auditory to tactile. Remarkably, in MSNs, the chunking pattern was unaffected by this task change and accompanying drop in performance accuracy; it continued to emphasize the beginning and end of maze runs despite the cue shift and the resulting robust shift in performance. Fast-firing neurons, putative striatal fast-spiking interneurons (FSIs), recorded simultaneously with MSNs, also developed the task-bracketing activity pattern. But the activity of these FSIs did change when the sudden task modification was introduced; they developed a phasic response at the onset of the new cue, a response that then faded with several days of further training.

These distinct MSN and FSI dynamics could be important for the striatum to maintain an overall structure of the task—a general plan or action set—while still processing changes in task details to adjust performance (Kubota et al., 2009). In other studies, the activity of striatal FSI populations has also been shown to relate to the suppression of unwanted or unselected movements (Wickens et al., 2007; Berke, 2011), suggesting that one important role of their sudden engagement at the T-maze cue shift could be to suppress pre-potent responses to the initial cue. They could effectively halt or segment the chunked behavior to permit flexibility in incorporating the new cue-response segment into a newly chunked behavioral pattern. Another important class of striatal interneurons, the cholinergic cells or tonically active neurons (TANs), show even further distinction in their dynamics related to task performance (Aosaki et al., 1994; Kimura et al., 2003; Apicella, 2007; Graybiel, 2008; Goldberg and Reynolds, 2011; Thorn and Graybiel, in press). The question of how interactions among these subtypes of striatal neurons relates to DLS function can now be addressed with cell-type specific markers.

### Dorsomedial striatum (DMS) plasticity tracks habit formation alongside dorsolateral striatum (DLS) stability

From the point of view of the field focusing on habit learning, a key issue for these T-maze experiments was the relation of the DLS task-bracketing pattern to activity in other striatal regions, particularly in the dorsomedial striatum (DMS). Inhibition of the DMS leads to a loss of behavioral flexibility and outcome-sensitivity, and an increase in habitual mode of responding, in multiple task conditions (Yin et al., 2005b; Ragozzino, 2007; Packard, 2009). These results were opposite to the results of inhibiting the DLS, suggesting that the two regions have opposing functions in relation to balancing flexible and inflexible behavior. If the DLS chunking pattern found in the T-maze experiments were related to habits, as suspected, then DMS activity recorded in parallel ought to be quite different, related to cognitive-associative components of the task rather than to habitual performance.

This prediction turned out to have support from the experiments of Thorn et al. (2010), who recorded simultaneously in both the DLS and the DMS while rats were trained on auditory and tactile versions of the T-maze task. The DLS formed its task-bracketing ensemble activity pattern early and maintained it as the animals proceeded through the extensive training protocol. Simultaneously, DMS ensemble activity during the decision time in the task increased as animals were learning—the time of lessened activity in the DLS. Then, as the animals became familiar with the task during over-training, this DMS decision-period activity waned. Thus a different set of dynamics marked habit learning in the two striatal regions. The DMS region in which these recordings were made was at about the same anteroposterior level as that of the DLS recordings, and so was anterior to the region studied in the loss of function studies (Yin et al., 2005a,b; Bradfield et al., 2013). A shift in the balance of DMS and DLS activity is emerging as a common marker of acquired habits in neural recording studies. For example, a recent experiment (Gremel and Costa, 2013) adapted a context-dependent habit task (Killcross and Coutureau, 2003) in which rats would behave habitually in one environment (in this case, where reward was delivered on a random interval schedule) but non-habitually in another environment (where reward was delivered on a random ratio schedule). Switching between habitual and non-habitual performance states in these environments was accompanied by a shift, in presumably the same neurons, between higher overall firing activity in DLS vs. DMS, respectively. Similarly, strengthening of DLS activity with decline of DMS activity has also been found in mice undergoing training on a rotarod task (Yin et al., 2009), suggesting this shifted medial-lateral balance in striatal activity also extends to skilled behaviors that were acquired through negative reinforcement.

The contrast in DLS and DMS neural dynamics suggests that, in a reinforcement context, changes in DMS activity might direct the expression of behaviors as they are being formed into habits, but once the DMS activity falls, then DLS-related circuits might take over the control of the behaviors, allowing them to be expressed as habit (Thorn et al., 2010). This notion meshes well with models derived from the results of lesion work, in which the rapid establishment of a habit following DMS (or prelimbic cortex) lesions is considered to reflect the uncovering of a DLS-associated habit that was dormant or occluded when the goal-directed system was intact (Killcross and Coutureau, 2003; Daw et al., 2005a; Yin and Knowlton, 2006; Balleine et al., 2009). In new work (Thorn and Graybiel, in press), evidence is emerging that theta-band oscillatory activity might allow the DMS and DLS to have different information flow through channels favoring sub-bands of theta. Moreover, in the T-maze task, which involves elements of both place and response learning, oscillatory local field potential activities in the striatum and hippocampus become progressively linked by the development of inverse phase relationships in the theta-band oscillations that they exhibit, especially during the decision-making parts of the runs (DeCoteau et al., 2007a,b; Tort et al., 2008).

If, as these studies suggest, there is parallel operation and possibly competition between circuits engaged by these striatal sub-regions in controlling behavior, then the brain might have a selection or arbitration mechanism for guiding the switch between them (Killcross and Coutureau, 2003; Daw et al., 2005a; Yin and Knowlton, 2006; Dolan and Dayan, 2013). The T-maze work suggested that the dynamics of the DMS activity might accomplish this function (Thorn et al., 2010), but left unresolved was which of the many DMS inputs might be driving the change, as well as the potential existence of similar habit-related neural dynamics in other brain regions. One potential route toward increasing or decreasing habit strength could be to enhance experimentally the decision-related DMS activity. However, the increase in strength of the bracketing pattern seen in some of these studies (Barnes et al., 2005) together with the increase in overall DLS activity that appears to occur as skills emerge in other studies (Tricomi et al., 2009; Yin et al., 2009; Gremel and Costa, 2013) raises the possibility that some task environments or types of learning might recruit late-stage plasticity in both DMS and DLS. As we discuss below, our evidence suggests that DLS is participating actively in action sequence expression and run-to-run automaticity of behavior at all stages, and is not only keeping the task-bracketing pattern ready for selection according to changes in the activity of other brain regions.

### Dorsolateral striatum (DLS) encodes action sequences and automaticity during an outcome-insensitive habit

Loss-of-function studies rooted in associative learning theory have largely been done separately from studies of the neural plasticity related to the formation of rewarded action sequences, but important attempts are being made to bridge the two research strategies (Tang et al., 2007; Kimchi et al., 2009; Stalnaker et al., 2010; Fanelli et al., 2013; Gremel and Costa, 2013; Smith and Graybiel, 2013a). In the T-maze, for instance, major questions remained about whether changes in neural activity, particularly in the DLS, related to habit formation as formally defined in outcome representation terms. Did maze performance reflect an A-O or place-based strategy, or an S-R or response-based strategy? Were dynamics of the DLS task-bracketing pattern related in any way to shifts between these strategies? How would S-R learning be reflected in the DLS dynamics?

To address some of these questions, we developed a modified version of the T-maze task in which a distinct reward was to be found at each of the two end-arms of the maze, and the reward-specific devaluation-sensitivity test was used to assay habitual behavior. Throughout this time, single-unit activity was recorded in both the DLS and its counterpart in the IL cortex, two canonical habit-promoting regions.

In the DLS, we observed the familiar task-bracketing pattern, with the firing of MSNs accentuating the run start, turn, and goal arrival. This pattern formed rapidly, around the time that animals reached a criterion of accuracy on the task. Importantly, however, parallel experiments in a set of control animals established that behavior was markedly goal directed at this early training point. In the probe test given on the day after devaluation, animals trained just to the learning criterion would immediately avoid the devalued goal (Smith et al., 2012). Thus, the DLS chunking pattern was emerging prior to habit expression proper. Once it formed, the DLS pattern remained steady during the over-training period, and, judging from the probe test given after the over-training period, the animals formed a devaluation-insensitive habit. After the probe test, when rewards for correct runs were returned, the animals’ behavior changed sharply; runs slowed, deliberative head movements increased, and accuracy dropped. However, as in the cue modality switch study discussed above (Kubota et al., 2009), the DLS pattern remained nearly unchanged. The DLS activity at the start, turn, and end of the runs remained as strong as it had been before. Behaviorally, animals were running correctly to the devalued goal (fewer trials) or to the non-devalued goal, or running the “wrong way” to the non-devalued goal when instructed to the devalued goal. At the session-wide level, it appeared that as long as animals were running a familiar route smoothly, whether the run was rewarded or not, the DLS ensemble pattern persisted. This possibility was supported further when we devalued all goals, which led to extinction of maze runs and a corresponding rapid loss of the chunking pattern (Smith and Graybiel, 2013a).

In trial-by-trial analyses of the DLS ensemble activity, and also in analyses of the activity of other subpopulations of DLS neurons, we did not find any clear relationship between the action-encoding in the DLS in any given trial and the timing of habit formation and suppression. DLS activity similarly lacked any correlation with sensitivity or insensitivity to outcome value on single runs. What we did find, however, was a remarkably close DLS relationship to the automaticity of a given run. During some runs, particularly early in training, animals would deliberate at the junction of the goal-arms, essentially conducting a “vicarious trial and error” by using their head to check each one before making a choice (Muenzinger, 1938; Tolman, 1948; Johnson and Redish, 2007). Both the strength of the DLS activity at the start of the run and the strength of the overall DLS chunking pattern were correlated inversely with these deliberative head movements: the DLS pattern was much stronger on trials that lacked this deliberation. This finding suggested that the DLS keeps track of or controls the level of automaticity or decisiveness of behavior at points of choice options. Much like the increased efficiency of movement-related signals that occurs in the DLS as actions are learned (Carelli et al., 1997; Jog et al., 1999; Graybiel, 2008), an increase in performance optimization appeared to characterize the buildup of DLS activity around major task actions. However, a particularly intriguing aspect of the results is that the major correlate of deliberative head movements occurred in DLS activity at the start of the run, and not in DLS activity at the turn itself when the deliberations were occurring or not (Smith and Graybiel, 2013a). Thus the activity was not related to the head movements themselves, but was anticipatory or predictive. This correlation of DLS activity with non-deliberative running was evident in early training as well as in late over-training, indicating a close involvement of the DLS run-to-run, more than just at the phase of behavioral learning when the behavior was expressed as a habit. In short, although the DLS pattern could mark the early formation of a habitual response plan (and “memory” of this plan after devaluation), it appears to relate quite closely to behavior on a trial-to-trial basis, even in demonstrably goal-directed performance phases.

## Implications for understanding habits: viewing dorsolateral striatum (DLS) activity as a starting point rather than a finishing line

### A puzzling disconnect between dorsolateral striatum (DLS) physiology and stimulus-response (S-R) behavioral theory

Some of these DLS dynamics seem surprising in the context of reinforcement learning models. As noted, there is a long tradition of referring to chained S-R associations as the underlying base of habitual behavior, and developments in learning theory have pinpointed conditions under which behavior might be understood as an S-R habit as opposed to an A-O behavior or Pavlovian conditioned response (Dickinson, 1985; Balleine and Dickinson, 1998; Daw et al., 2005a; Yin and Knowlton, 2006; Holland, 2008; Schneck and Vezina, 2012). The DLS is widely regarded as a source, if not *the* source, for such associations in the brain. Yet fitting aspects of the neural recording data within this framework is not straightforward. Concerning our maze task alone, we have highlighted the many dynamic processes that exist in DLS physiology, including those of different types of task-related patterns, different task epochs, and different neuronal types. Notable for this discussion is the finding that the DLS pattern forms prior to habit emergence, correlates with how automatic a run is from early on, and remains stable after devaluation despite major accompanying changes in behavior and valuation of stimuli, responses, and outcomes. Moreover, we, as our colleagues, find relatively few neurons in the DLS that have preferential responses to S-R combinations (Berke et al., 2009; Thorn et al., 2010; Smith and Graybiel, 2013a). One complex way that these findings might be reconciled with an S-R view is if behavior were partly habitual from the moment of DLS pattern formation (which it might be), and if that particular habit memory were stored stably and used selectively during over-training but not used during most runs after reward devaluation.

Contrasts have also arisen in related work showing that as a devaluation-insensitive habit develops, there is a loss of specific head-movement-related activity in DLS neurons in a head-bobbing task (Tang et al., 2009). However, a similar weakening of neural activity of tongue-related units in lateral striatum occurs with training on a licking task, but this licking behavior remains devaluation-sensitive (Tang et al., 2009). In another study, action-related neural responses that are modulated by the identity of a preceding cue arise in the DLS (Stalnaker et al., 2010). Although this result would appear to offer evidence for an S-R association, the same correlates arise simultaneously in the DMS, a “non-habit” site. The authors speculate that S-R integration was occurring elsewhere, perhaps in sites receiving DLS outputs. All of these incongruities might be good indicators that we should look elsewhere than in the DLS or its outputs for habit-related activity. However, if the neural dynamics that dominate DLS recordings in these studies are considered to reflect important features of ongoing behavior, as they certainly appear to, different conclusions arise. One is that stimulus-specific response plans might be supported more widely in the striatum than we thought. Another is that the increased efficiency of performance-related representations in DLS activity might aid behavior in a wide variety of Pavlovian, goal-directed, and habitual tasks. And as is so often true, the specific demands of tasks shape the patterns of activity found.

Returning to the T-maze, the findings that we review suggest that the DLS can contribute to habits in a way that does not encode a chain of task-related S-R associations, a possibility that has at least some support in the literature. Although S-R associations need not take any particular form in neural activity, the lack of stimulus-evoked firing in the DLS has raised the question of how habits are represented in this region if not by responses to paired Ss and Rs (difficult-to-isolate contextual S’s notwithstanding) (Berke et al., 2009; Root et al., 2010; Thorn et al., 2010; Smith and Graybiel, 2013a). Similarly, although abolishing dopamine input to the DLS leads some behaviors to be devaluation-sensitive (i.e., not “habitual”) (Faure et al., 2005), a task designed to tap into S-R learning strategies can be performed mostly normally in Parkinsonian patients with similar dopaminergic dysfunction, suggesting to the authors an independence of S-R learning from the DLS (de Wit et al., 2011). From the brain’s point of view, the neural dynamics that do occur during habit formation might provide some insight into the role of DLS in mediating behavior, even if it does not lead precisely to the same S-R notions that inspired the research.

### Dorsolateral striatum (DLS) action chunking as habit formation

The dominant pattern that emerges in the DLS during habit formation, at least on the maze, is the accentuation of salient actions or behavioral boundaries and has a close relationship to run automaticity. If we take this pattern as a starting point, action chunking seems like a plausible underlying DLS mechanism for controlling habits (Graybiel, 1998). In this way, sequences of actions can become linked and can be executed as a unit automatically or semi-automatically. To the extent that associations are driving the chunking process, the links that are made between each action could provide a means for dissociating behavior from the outcome.

The chunking together of the actions and accentuation of start-related activity can be viewed as enhancing the performance of the bounded sequence of actions, once the sequence is initiated. This is an important property, one that could permit actions even close to reward to be conducted in the context of the chunked-together unit. There is much evidence that under normal conditions, actions and cues that are proximal to reward are more closely associated with its specific features, and thus are more sensitive to shifts in its value or contingency. Actions or cues occurring more distally to reward might carry a more diluted reward representation arising from additional associations made with subsequent actions and cues, yielding more outcome-independence. This touches on the problem of credit assignment in reinforcement learning theory (Sutton and Barto, 1998). Empirically, although reward-distal cues appear to make good predictors of impending reward, they are less sensitive to shifts in motivational state or outcome value compared to reward-proximal cues (Tindell et al., 2005; Zhang et al., 2009; Smith et al., 2011). Conditioned behavioral responses to cues, instrumental actions, and species-specific behaviors (e.g., predation in the cat) show a similar sensitivity to state or reward value changes based on proximity to actual reward receipt (Morgan, 1974; Holland and Straub, 1979; Balleine et al., 1995; Holland et al., 2008).

This phenomenon seems to bear out in the maze as well. When we devalued all rewards on our maze after the initial devaluation, the DLS pattern was rapidly lost. The rats showed a progressive backward breakdown effect (Morgan, 1974), first failing to drink the reward and then failing to turn upon instruction, and yet persisting in the initial run initiation (Smith and Graybiel, 2013a). This result raises the possibility that the maze running actions, once released from being expressed as a chunk, could show similar variations in flexibility based on their proximity to reward. By extension, when previously chunked as a sequence, the full run behaved like the most reward-distal action (i.e., run start). This proposal meshes quite well with the finding that devaluation of a well-learned action sequence can lead to the loss of the sequence in full, as opposed to the loss of only the reward-proximal element (Ostlund et al., 2009). Similarly, monkeys trained to press buttons sequentially in order to obtain reward will continue to conduct the full sequence even if the reward is made available to them earlier, an effect that appears to relate to the strength of striatal dopamine input (Matsumoto et al., 1999). Similar continuation of reward-seeking acts despite disinterest in the reward itself has been noted in a variety of older behavioral studies (see Morgan, 1974). This general phenomenon calls to mind the famous “kerplunk” effect (Carr and Watson, 1908), in which animals are trained well on a complex maze and then an experimenter suddenly moves the reward (and end-wall) to a closer position. Well-trained animals continue running right past the reward and contact the wall, as though they had formed a habit of a certain response set that would be carried out in full even if it resulted poorly.

We raise the possibility that part of this action-chunking and outcome-decoupling process might involve a motivational value that is bound to the chunked action sequence itself. Cues, for instance, can grow to acquire incentive value specific to the reward with which they are paired; the cues then become attractive and meaningful, and can pull in behavior (Bolles, 1972; Bindra, 1978; Toates, 1986; Berridge, 2004; Rudebeck and Murray, 2011). Something similar might occur with strongly reinforcement, by which doing them becomes attractive and rewarding in its own right (Berridge, 2009). Glickman and Schiff (1967) suggested that some actions, such as those related to food consumption or copulation, have had value attached to them over the course of evaluation. DLS activity might be one candidate substrate for this value-binding process. Such a view might help to account for several observations: the strong accentuation of reinforced actions by DLS activity in the T-maze task; the fact that this activity pattern was maintained after devaluation of one reward (i.e., when animals mostly ran to the non-devalued goal regardless of instruction, as though it were an immediately valued route); and the fact that the activity pattern was lost after all outcomes were devalued and behavior was extinguished (when values decay). Actions attaining incentive value could in principle drive behavior independently from expected outcome value, and thus function much differently than the “action value” signals of reinforcement learning and behavioral economics (Rangel et al., 2008).

This action-bound value conceptualization of chunked behaviors might ultimately also help link together disparate functions of the DLS, including its contribution to behaviors that are not easily interpreted in the context of prediction-error learning, such as instinctive grooming patterns and Pavlovian-to-instrumental transfer (Aldridge et al., 2004; Corbit and Janak, 2007). Why is there an impetus to perform such actions? There are many possible reasons, but one could be that the behaviors have an intrinsic incentive motivational value, whether innately expressed or acquired through experience. More broadly, too much or too little value might contribute to excessive drive to perform actions (as in obsessive-compulsive spectrum disorders) or loss of the capacity to perform intended actions (e.g., in Parkinson’s disease).

Many of the relationships between DLS activity and habitual behavior that we have emphasized are at the level of correlations. Similarly, the lack of detection of robust S-R or other representations in the DLS is not evidence that they are not present; chunking-related activity is a dominant DLS signal, but S-R encoding could well be found in other tasks, in other brain regions, or in other features of DLS physiology. Even how the task-bracketing pattern relates to the motor demands of the behavior has not been fully documented. We especially emphasize that in the DLS, the relatively low activity in-between the pronounced beginning and end of the runs does not mean a lack of activity at these times. Further “expert neurons”, with highly specialized functions, remain active even during mid-run when ensemble activity quiets (Barnes et al., 2005; Thorn et al., 2010).This pattern suggests that sparse coding, a feature of many neural systems, could be built into the DLS activity, reflecting the capacity for a few neurons to encode the full sequence, and/or that after a behavioral sequence has become habitual, it is advantageous to free up neurons to participate in other computations. Finally, as originally discovered by Barnes et al. (2005), the chunking pattern is shown by a large population of DLS projection neurons, but many other neurons become quieted throughout the runs as the maze training proceeds. It is still not clear whether the quieted neurons form a special class, for example, the so-called indirect pathway neurons as opposed to direct, as discussed by Thorn et al. (2010). If so, this distinction would parallel the superficial layer-deep layer dichotomy in the bracketing pattern observed in the IL cortex (Smith and Graybiel, 2013a). Clearly further research is needed to characterize the control mechanisms of action chunking in the striatum and elsewhere (Graybiel, 2008; Desmurget and Turner, 2010; Dezfouli and Balleine, 2012). Thus far, however, evidence is compelling: (1) that habits are, in part, chunked action sequences; (2) that this function is reflected, in part, in dynamic patterns of activity in the DLS and DMS; and (3) that action sequence chunking, as reflected by this activity patterning, is a main, but not sole, contribution of the DLS to habits.

## Dual operators for habits: contrasting dynamics in the infralimbic cortex (IL) and dorsolateral striatum (DLS)

The prefrontal cortical region known as IL cortex has been identified as having habit-promoting functions similar to those of the DLS, despite its apparent lack of direct connections with the DLS (Figure 1). Lesions or inactivation of the IL cortex result in outcome-sensitivity and habit blockade (Coutureau and Killcross, 2003; Killcross and Coutureau, 2003). However, there are specific details of the tasks employed in these studies that are important to consider. Normally, animals trained to the point of exhibiting habitual behavior on a lever press task will continue pressing both a lever paired with a reward that has been devalued and a lever paired with a non-devalued reward. Lesions or GABAergic inhibition of the IL cortex lead animals to press more than normally on the lever for the non-devalued reward, but the animals do not increase pressing on the lever for the devalued reward (Coutureau and Killcross, 2003; Killcross and Coutureau, 2003). These results provided evidence of a more goal-directed form of behavior that would otherwise be habitual were the IL cortex intact. Intra-IL microinjection of dopamine similarly leads to increased pressing on a lever for a non-devalued reward, but it also concomitantly reduces pressing on a lever for a devalued reward (Hitchcott et al., 2007). Collectively, these findings identify the IL cortex as a prefrontal region that is important for maintaining an outcome-insensitive habit. The distinct result of increased behavior to a valued goal following IL disruption has led to the suggestion that the IL cortex (by way of being affected by dopamine levels) might control changes in the allocation of both goal-directed and habitual strategies when outcome value changes (Hitchcott et al., 2007; Dolan and Dayan, 2013).

To evaluate the extent to which habit formation might be reflected similarly in the firing patterns of the IL cortex and the DLS, we recorded in these regions simultaneously as we trained series of rats in the two-reward maze paradigm (Smith and Graybiel, 2013a). We found that the bracketing pattern developed in the IL cortex as well as in the DLS, but that its formation was delayed until well into the over-training period. This finding, and the differential sensitivity of the IL cortex and DLS patterns to reward devaluation, raised the possibility that the IL cortex and the DLS function as distinct core contributors, or operators, in the development of habits. In this view, habits are promoted by at least two underlying controllers in the brain. The dynamics we recorded suggested a mixed redundancy and distinctiveness of the activities in the two regions, and provided information about the circuit-level patterning of neural activity that occurs as habits are made.

The similar action-bracketing activity in the IL cortex and DLS suggested the existence of at least one common neural signature of habit formation shared by IL-associated prefrontal and DLS-associated neural circuits. The IL pattern was slightly different in terms of makeup, being more broadly activated just prior to run initiation and prior to goal arrival, but in the DLS, as in the IL cortex, activity during the decision period was greatly diminished. The timing of the changes in activity patterns in the two regions was strikingly different. IL activity scarcely changed until the late over-training period, when our behavioral devaluation measures had shown was the time that the maze habit became crystallized, shifting from being devaluation-sensitive to devaluation-insensitive. At this time, the IL task-bracketing pattern formed and persisted until reward devaluation. Although the IL pattern was present during the probe test, at which time behavior was expressed as habitual, it rapidly decayed when the rewards were returned in post-probe training sessions and running choices changed. Later, after a prolonged period of continued post-probe training, the IL pattern returned. This period of further plasticity in IL activity corresponded to a stage at which a second, replacement habit probably had formed on the maze, given the behavior of the rats: initially after devaluation, they began to avoid the devalued goal and, instead, began to run to the non-devalued side. These wrong-way runs increased in frequency over days (despite lack of reward for them), became faster and increasingly similar to the instructed runs to the same location, and lost the deliberative head movements at the turn that had appeared earlier. Thus, the return of the IL task-bracketing pattern appeared to mark the formation of a new habit of simply running to the non-devalued goal. We suggest as a leading possibility that the alignment of the more flexible IL chunking pattern of activity to the more rigid chunking pattern expressed in the DLS might be necessary for expressing behavior as a habit.

Unlike the ensemble activity in the DLS, the IL activity pattern could not be linked consistently to any run-to-run variation of behavior that we assessed. This negative finding was surprising, as it seemed to suggest that the IL cortex was not functioning in our task as an arbiter or habit-selector as had been speculated. If the IL cortex had such a function, its activity ought to reflect, on a given trial, whether behavior was outcome-sensitive or outcome-insensitive, deliberative or non-deliberative. This form of coding has been observed in other cortical regions (Wunderlich et al., 2012), and is essential for allowing a rapid toggling between more goal-directed and habitual strategies in some ongoing behaviors (Dolan and Dayan, 2013). We also might have expected an outcome value-tracking signal if the IL cortex were, at some level, aiding in goal-directed behavior as prior work suggested (Hitchcott et al., 2007). What we found, instead, was that the IL task-bracketing activity was inversely correlated with the net number of deliberations and level of outcome sensitivity that occurred in entire sessions, each composed of many individual maze runs. The contribution of IL cortex to the maze running habit might therefore be at the level of a state property (Smith and Graybiel, 2013a). Much as the state of stress can lead to either cribbing or pacing in horses, here the state contributed by IL activity could promote habits without specifying their behavioral details.

The task-bracketing ensemble activity that we observed in the IL cortex was mostly restricted to neurons located in the superficial layers of the IL cortex. Neurons recorded from deeper layers exhibited plasticity in firing rate at nearly identical time points, but during runs, the deep layer ensembles became active throughout the run. Thus, there was striking layer-selectivity to the habit-related activity patterns. An interesting possibility is that the chunking pattern in IL cortex is indicative of activity that is communicated across trans-cortical networks; but we lack enough information to test this possibility. We do point out that the bracketing pattern was not general to the prefrontal cortex. Ensemble activity recorded from the overlying prelimbic cortex declined as the habit emerged, consistent with its DMS-like role in promoting behavioral flexibility (Balleine and Dickinson, 1998; Killcross and Coutureau, 2003).

These findings suggested a far more central role for the IL cortex in habit acquisition and expression than formerly appreciated. By some views, the IL cortex was thought to serve as an arbitration or contention-scheduling mechanism for selecting goal-directed vs. habit strategies (Coutureau and Killcross, 2003; Daw et al., 2005a), or to promote habits by acting on learned associations stored elsewhere (Balleine and Killcross, 2006). The dense connectivity that the IL cortex shares with limbic and associative regions, including the amygdala and ventral striatum (Hurley et al., 1991; Vertes, 2004), suggests that IL activation feeds forward to dampen evaluative processes in these sites or to invigorate motivation or prior learned associations in order to promote behavioral persistence. The development or decrease of IL activity—possibly specifically of its task-bracketing activity—might ultimately provide a permissive state for habits.

Much like the weakening of DMS activity that we have seen during over-training on the maze (Thorn et al., 2010), the timing of IL plasticity could similarly be critical for habits—when the task-bracketing pattern is expressed alongside the similar ensemble pattern in the DLS, behaviors grow outcome-insensitive. Ultimately, the stages of plasticity in the IL cortex, in the DMS and in their associated circuitries might thus determine or allow the full strength of a habit to be expressed, the essential involvement of DLS in action sequencing or valuation notwithstanding (Killcross and Coutureau, 2003; Yin and Knowlton, 2006; Balleine et al., 2009; Thorn et al., 2010; Smith and Graybiel, 2013a). Research from several laboratories has begun to identify the neurochemical/molecular signals within IL cortex that might aid this privileged function, including transmission involving GABA and dopamine (Coutureau and Killcross, 2003; Hitchcott et al., 2007; Barker et al., 2013).

The neural dynamics and behavioral correlations uncovered in the T-maze study suggest an intimate participation by the IL cortex in sculpting and maintaining habits in addition to a role in selecting them (Hitchcott et al., 2007; Smith and Graybiel, 2013a). If so, it should be possible to test, by means of optogenetic interventions, the view that the IL cortex contributes core components to a habit, just as does the DLS. We have done such testing in two ways.

## Optogenetic interventions to test the impact of real-time activity dynamics in the infralimbic cortex (IL)

### Roles of the infralimbic cortex (IL) in habit expression

To evaluate a causal contribution of ongoing, real-time activity in the IL cortex to habitual behavior, we incorporated an optogenetic approach (Smith and Graybiel, 2013b). The spatiotemporal resolution provided by optogenetics, now widely noted (Boyden et al., 2005; Bernstein and Boyden, 2011; Fenno et al., 2011; Mei and Zhang, 2012), allowed us to restrict IL disruption to particular time windows of specific populations of neurons. We sought to perturb IL activity only during the maze runs, not before or after the runs, in order to evaluate on-line functions of IL cortex pyramidal neurons during behavior (Smith and Graybiel, 2013b).

We first examined the effect of halorhodopsin-mediated perturbation of IL pyramidal cell activity in over-trained rats, testing during the probe session after they had been given reward devaluation (Smith et al., 2012). Control rats lacking this perturbation behaved like normal over-trained rats: they continued to run, “by habit”, to the devalued goal as well as to the non-devalued goal. Rats with IL inhibition, however, exhibited outcome sensitivity: they reduced their running to the devalued goal when so instructed, by about 50%, and thus behaved like rats that had not received the over-training. This effect replicated the ability of IL lesions or chemical treatments to block habitual behavior (Coutureau and Killcross, 2003; Killcross and Coutureau, 2003; Hitchcott et al., 2007), showing again that removing the influence of IL cortex over behavior suppresses actions directed to a devalued goal and shifts them to a more valued one. The optogenetic approach further added a critical fact: that IL cortex exerts powerful on-line influence over ongoing behavior. Avoidance of the devalued goal occurred within a few trials, amounting to just seconds of IL inhibition time. Moreover, rats persisted in avoiding the devalued goal on subsequent days without further inhibition, and did not rebound or have to “learn again” when the inhibition was removed; the habit blockade effect endured. Additional IL perturbation at this stage did nothing to behavior: animals continued to show elevated running to the non-devalued goal and decreased running to the aversive goal, suggesting that IL activity during runs might not be necessary in some contexts for expressing this outcome-appropriate strategy of behavior (Hitchcott et al., 2007; Smith et al., 2012; Dolan and Dayan, 2013).

A major surprise then came when we gave another round of IL perturbation after the prolonged post-devaluation training. At this stage, IL neural activity had begun to exhibit the task-bracketing pattern again, and behavior appeared to reflect the emergence of a second wrong-way running habit. When we applied IL perturbation at this time, but not earlier, this second behavior (putative replacement habit) was blocked with similar immediacy. The frequency of wrong-way runs suddenly decreased, and rats ran back to the devalued goal when so instructed (and drank the reward), whereas control rats continued their pattern of wrong-way runs. This behavior again endured over subsequent sessions, and further sessions of IL perturbation almost entirely returned rats to their originally learned behavior, tested for up to 20 days after the habit reinstatement first occurred.

During these striking optogenetically induced changes in the rats’ behavior when instructed to go to the devalued goal, the IL perturbation had no detectable effect on the instructed runs to the non-devalued goal at which the normal reward could be found, as cued. Nor did it affect home-cage intake of the devalued reward where the aversion persisted even in the presence of IL inhibition. These results therefore suggested a highly specific impact of the IL perturbation on the maintenance of acquired habitual strategies of running. The IL cortex thus does appear to have on-line control over habitual behaviors as they occur. This on-line control system exerts a remarkably rapid, robust, and enduring influence over behavior, likely reflecting rapid plasticity in downstream targets of the IL cortex.

Why did the blocked habit come back when the IL cortex was later perturbed? One possibility is that the IL cortex maintains *newly* formed habits, at least for a time. If the task-bracketing pattern, when present, were to contribute a state in which a new response strategy can be executed habitually, as in situations in which it competes with an alternative strategy, then blocking this IL pattern after initial over-training would return rats to a prior strategy, namely, outcome-sensitive or exploratory running. Blocking this pattern again later, after the second habit formed, would similarly strip this second habit away, and then the prior strategy—the initial habit—would be expressed. By this account, the IL does not store initial habits when new ones arise, but does contribute critically to promoting the most situation-appropriate ones that have been learned.

This interpretation could carry implications for considering IL function more broadly as it relates to regulating other learned behaviors. Famously, the IL cortex regulates extinction learning across a range of tasks (Morgan et al., 1993; Rhodes and Killcross, 2004; Peters et al., 2009). The IL cortex also participates directly in maintaining new strategies in tasks requiring animals to shift between using spatial cues vs. response plans to perform (Ragozzino, 2007; Rich and Shapiro, 2009). Depending on conditions, IL inhibition can also lead to a spontaneous recovery of an extinguished drug-seeking behavior, or can conversely prevent the return of drug seeking that would normally be evoked by exposure to a drug context (Peters et al., 2008; Bossert et al., 2011). One possibility is that the IL cortex is specialized for promoting a new response strategy at the expense of an older, prepotent one—be it a new habit, a new response inhibition, or a new mnemonic strategy (Killcross and Coutureau, 2003; Rich and Shapiro, 2009; Smith et al., 2012). The many output connections of IL cortex could support the translation of this general function into different behavioral effects in different situations (Peters et al., 2009). Our finding that IL inhibition could both block and reinstate a particular habit certainly suggests some form of dependency of IL function on context or history (Smith et al., 2012).

### The activity of infralimbic cortex (IL) during habit acquisition

If IL cortex is critical to the on-line expression of habits, it could be critical also for the acquisition of habits, given a generalized form of on-line monitoring or control by this part of the prefrontal cortex. To test this possibility, we asked whether optogenetic perturbation of IL cortex could prevent the formation of a new habit (Smith and Graybiel, 2013a). An advantage of gene-based targeting approaches, as opposed to lesions or drug microinjections, is that cell populations can be manipulated repeatedly without compromising the integrity of tissue. Leveraging this strength, we tested, albeit with imperfect layer- and cell-type-specific manipulations, whether the IL task-bracketing pattern during the over-training period—the time during which we had found this pattern to form in conjunction with habit formation—was critical to the crystallization of the maze running behavior as a habit. We applied halorhodopsin-mediated perturbation to the IL cortex on each day during over-training on the T-maze task. Animals then underwent reward devaluation and a post-devaluation probe test without further IL inhibition. The behavior of the animals during the probe test clearly showed that they had not formed a habit despite being over-trained. The rats with IL inhibition during the entire over-training period acted like rats with no over-training experience: they avoided the devalued goal during the probe task, behaving as though they had only received initial training to criterion, but had not been over-trained. This finding demonstrates that the IL cortex contributes to more than just habit selection—on-line IL activity during performance is essential for making habits in the first place, as well as for expressing them once they are formed.

This finding also underscores the potential of using temporally precise manipulations for affecting even strongly ingrained and multifaceted behaviors, such as habitual behaviors, as well as for testing causal roles of particular on-line neural dynamics (Boyden et al., 2005; Bernstein and Boyden, 2011; Fenno et al., 2011; Smith and Graybiel, 2013b). Related work on corticostriatal systems and action selection has put optogenetic and pharmacogenetic approaches to use in isolating pathways and cell types necessary or sufficient for goal-directed behaviors (Gremel and Costa, 2013), ritualistic behaviors (Ahmari et al., 2013; Burguiere et al., 2013), behavioral initiation or cessation (Kravitz et al., 2010), and linking rewards or drugs with behavioral plasticity (Witten et al., 2010; Ferguson and Neumaier, 2012; Kravitz et al., 2012; Stuber et al., 2012; Lenz and Lobo, 2013). As we have noted (Smith and Graybiel, 2013b), research on habits will benefit tremendously from continued work with these methods and their successors.

## Continued reorganization of habit research?

### Unfinished business: brain mechanisms

Research is not even close to resolving mechanisms underlying habitual behavior, even work on the DLS, toward which the most attention has been paid. The striatum contains multiple subtypes of neurons (and subtypes of glia) and complex sets of inputs; and only recently have we been able to study the dynamics of these components in relation to behavior. Even aside from behavior, there is rapid progress being made at every level of analysis of striatal activity; conceptually, each discipline is like holographic representation of the larger technological revolution that is affecting neuroscience research directions. In striatal physiology, for example, such technology is producing surprises. For example, co-release of classical neurotransmitters and neuromodulators, such as glutamate, GABA and dopamine, and complex interactions among interneurons and projection neurons have been identified *in vivo* within the striatum (Stuber et al., 2010; Tecuapetla et al., 2010). As a second example, stimulation of cortical excitatory, glutamatergic inputs can lead to predominant suppression of MSNs in a behaviorally relevant manner, due to effects of the cortical afferents on GABAergic interneurons (Burguiere et al., 2013). So much for the plus and minus signs in our diagrams!

Given this rapidly changing face of models of striatal organization and function, it is unclear how the neural recordings that we and others have made of the striatum during habit learning relate to underlying mechanisms. Striatal MSNs are divided into the D1-receptor expressing striatonigral (direct) pathway and D2-expressing striatopallidal (indirect) pathway (Alexander et al., 1986; Albin et al., 1989; Graybiel, 1995). Additional compartmentalization occurs through the striosome vs. matrix organization of MSNs, and in the “matrisome” input-output organization of cortico-basal ganglia circuitry (Graybiel, 1995; Crittenden and Graybiel, 2011). Recorded neurons do not fall cleanly into categories that, as yet, map on to this anatomical organization; there are non-task-related neurons, task-related neurons, reward-related neurons, and multiple subtypes within each group. Making sense of this physiological complexity in terms of anatomical connections, as well as in terms of intrastriatal neuronal interaction, will be essential for progress toward understanding habits even just at the level of mechanism in DLS firing dynamics.

Similar points can be made of the IL mechanism driving habitual behavior. Given the heterogeneity of cell types, laminar organization, the multitude of inputs and outputs, and the stunning line of studies now coming out revealing the true complexity of it, much remains to be uncovered. How the IL cortex interfaces with, or does not interface with, the DLS at different phases of habit learning and expression is another major focus of interest. Progress can be now made in comparing stimulations and inhibitions of different pathways or cell classes in either site, or comparing these manipulations at different points in time, as it relates to action and habit learning. What habits are, as the brain sees it, is likely to grow more complex and interesting.

Finally, extending this work to the domain of human behavior will be important. While the DLS shares anatomical homology with primate putamen (or primate DLS) and some evidence indicates a similar functional homology for habits (Tricomi et al., 2009), it is unclear what the primate correspondent of the rodent IL cortex is. The IL cortex shares some functional similarity with the human ventromedial prefrontal cortex in relation to fear extinction (Milad et al., 2006), and to Broadmann Area 25 in relation to aspects of anatomical connectivity and role in depression symptomology (Covington et al., 2010; Holtzheimer and Mayberg, 2011). However, concerning habits, essentially no links have yet been established.

### Unfinished business: outcome-sensitivity measures and the instrumental/Pavlovian distinction

There is similar opportunity for progress in characterizing habits at the behavioral level. The outcome devaluation test has become a standard for defining behaviors as habitual (aside from tests of A-O contingency), and it has become common to think of performance that fails to meet this outcome-sensitivity measure as an S-R habit. However, there are important qualifications to consider. Experiments on Pavlovian learning, for example, have shown that the way in which reward is revalued (e.g., high-speed body rotations vs. taste aversion), and the type of conditioned response being evaluated (e.g., orientation to cues vs. food approach and consumption), can matter greatly for measurements of outcome sensitivity (Morgan, 1974; Holland and Rescorla, 1975; Holland and Straub, 1979; Galarce et al., 2007; Holland and Wheeler, 2009). One fascinating example, sign-tracking or auto-shaping (approaching a cue rather than the source of the reward that it predicts), can in some conditions appear to be insensitive to outcome associations and habit-like (i.e., resistant to an omission contingency) (Stiers and Silberberg, 1974; Hershberger, 1986), whereas in other conditions it can appear as outcome sensitive and quite non-habitual (Locurto et al., 1976; Robinson and Berridge, 2013).

There are also compelling conceptual accounts of behavioral persistence despite changes in outcome value that do not refer to an underlying S-R mechanism. For example, Tolman suggested such persistent behavioral “fixation” resulted from overly strong “sign-gestalt” knowledge acquired in an environment (Tolman, 1932, 1948), Berridge conceives of reward cues as gaining an incentive value that can become “defocused” or detached from moment to moment changes in predicted reward value (Berridge, 2012), and Holland highlights the principle that outcome representations during behavior can become generalized (i.e., something good) vs. being percept or identity specific (i.e., chocolate milk), and that in such former instances they can simply lose their associability with the reward (Konorski, 1967; Holland, 2004; Holland and Wheeler, 2009). Thus, sensory features surrounding major action events on any task could potentially instill devaluation-insensitivity through non-instrumental means, including in our T-maze task. However, the clear action-related DLS/IL activity dynamics support action control as the most parsimonious explanation for our findings. In the broader neuroscience field, strong clues are coming from work on stereotypic or repetitive behaviors, some of which have been shown to result from specific genetic mutations in genes expressed in the striatum and elsewhere (Berridge et al., 2005; Welch et al., 2007; Burguiere et al., 2013) or in specific pathways (Canales and Graybiel, 2000; Hyman et al., 2006; Pascoli et al., 2011; Milad and Rauch, 2012; Ahmari et al., 2013; Burguiere et al., 2013). Such ongoing research at the mechanism level is sure to help us better understand how persistent behaviors arise, and the ways in which they can or cannot be understood as habits. Similar considerations apply to addictive behaviors.

### Complementary behavioral measures

Additional measurements aside from outcome manipulations can be used to indicate that behavior on a task has become habitual, but it is not always clear how they go together. In the T-maze studies, we observed a progression of behavior toward outcome-insensitivity, loss of deliberations, and increase in run speed and accuracy (Smith and Graybiel, 2013a). Yet, on a trial-to-trial basis, these measures were not necessarily aligned. Animals might deliberate at a turn but then approach a clearly devalued goal. Or they might avoid a devalued goal, yet lack any sign of deliberative movement. Similarly, we noted that animals slowed demonstrably in run speed during the unrewarded probe session, and yet they still displayed total insensitivity to outcome value and performed accurately; thus, they were behaving by “habit”, even though their behavior was slow and partly extinguished. Much evidence suggests that outcome sensitivity is a better indicator of habitual behavior than accuracy or speed to define a habitual response (Balleine and Dickinson, 1998). But we need to gain better ways to distinguish habits and skills. For the habits we have studied, defined on the basis of their insensitivity to reward devaluation, multiple behavioral parameters seem to reflect different structural features of the habits. Habits, according to our observations, can be compound behaviors, and in their development can display different components and have mixed characteristics, for example, being deliberative but outcome-insensitive. The breadth of the activity patterns that we have encountered in making multiple recordings from the striatum and medial prefrontal cortex suggest such compound features as well. Further, we have not touched upon the insistent, extreme habits and repetitive behaviors that can arise from exposure to drugs or appear within the context of neurologic and neuropsychiatric disorders. Searching for a single definition of habitual behaviors may be less productive than searching for the multiple potential circuit-level mechanisms that lead to habits, skills and repetitive behaviors. Finally, we have not approached the mnemonic aspects of habitual representations. These are of the greatest interest to explore.

### Integration with computational approaches

Computational work related to habit formation has also moved away from thinking of habits purely in terms of chained S-R reflexes, favoring instead contrasting behaviors based on stored state-based action values learned through prediction-error mechanisms (i.e., model-free behavior) with model-based exploratory behavior (Daw et al., 2005a; McDannald et al., 2012; Dolan and Dayan, 2013). Incorporating the dissociability of outcome and deliberation-related measures of habits together with performance optimization measures might be of particular interest in formulating the underlying rules of behaviors that have model-free components. Habits, considered as sequences of actions, can also, as argued by Dezfouli and Balleine (2012), be captured by model-*based* learning rules. This kind of debate underscores the reorganization that is occurring in our thinking about habits at many levels.

In the context of our own work, we suspect that the IL cortex and DLS constitute just two of multiple control systems in the brain, and hierarchical computational models need to be, and are being, developed in this domain. Yet, at the heart of the dual-operator notion that we present for these two regions is the idea that they have controlling power in shaping outcome-insensitive and non-deliberative action sequences, distinct from other co-existing systems important for reward valuation and performance learning. The neural dynamics, in particular the chunking pattern of activity with dissociable time-courses of plasticity and relationships to behavior, suggest that these two regions and their associated circuits together allow the crystallization of behaviors into chunked action-plans that can be executed semi-autonomously despite ongoing changes in the external world.

## More to come in exploration of habits as a model for studying the balance between flexible and fixed behaviors

We have taken the brain’s perspective in this review, and have suggested that habits are composed of multiple operators, two of which are reflected in the neural activity dynamics of the medial prefrontal cortex and dorsolateral part of the striatum. Evidence from this work indicates a relation of the striatal DLS to action chunking and the level of automaticity of the habitual behavior, and a relation of activity in the IL region of the medial prefrontal cortex, especially its upper layers, to promoting the formation and expression of chunked behaviors as outcome-insensitive and non-deliberative. If we set aside the historical notion of S-R chains as the sole defining features of ongoing habits (arguably premature for some habits), we make room for considering flexible dynamics of neural activity across multiple brain circuits and their microcircuits as providing the neural structure underlying habits. We have only touched on the functions of a small set of regions implicated as being necessary for habit formation, or necessary for habits not to be formed, as animals explore their environments. We make the point, however, that the degree of on-line control over habits by small regions of the medial prefrontal cortex is remarkably strong, and we make the further point that the cooperative activity of regions that were once thought of as acting in opposition actually could be at the heart of the capacity to form enduring habits, behaviors that are of great value in our lives.

## Conflict of interest statement

The authors declare that the research was conducted in the absence of any commercial or financial relationships that could be construed as a potential conflict of interest.
